# Pathological fracture due to primary bone lymphoma in a patient with a history of prostate cancer: A case report and review of literature

**DOI:** 10.3389/fonc.2023.1100559

**Published:** 2023-03-16

**Authors:** Pooja Bhakta, Zachary E. Hunzeker, Juan D. Garcia, Ayman Youssef, Bradley J. Grant, Rasha Alfattal, Dylan Weaver, Peeyush Bhargava, Ariel Rischall, Tejo Musunuru, Palawinnage V. Muthukumarana, Jayati Mallick, Kirill A. Lyapichev

**Affiliations:** ^1^ Department of Internal Medicine, John Sealy School of Medicine, University of Texas Medical Branch at Galveston, Galveston, TX, United States; ^2^ John Sealy School of Medicine, University of Texas Medical Branch at Galveston, Galveston, TX, United States; ^3^ Department of Pathology, John Sealy School of Medicine, University of Texas Medical Branch at Galveston, Galveston, TX, United States; ^4^ Department of Radiology, John Sealy School of Medicine, University of Texas Medical Branch at Galveston, Galveston, TX, United States; ^5^ Division of Hematology and Oncology, Department of Internal Medicine, John Sealy School of Medicine, University of Texas Medical Branch at Galveston, Galveston, TX, United States

**Keywords:** primary bone lymphoma, diffuse large B cell lymphoma (DLBCL), femur, diaphyseal fracture, pathological fracture, rituximab, CHOP

## Abstract

Primary bone lymphoma (PBL) is a rare extranodal presentation within lymphomas and primary bone malignancies. Pathologic fracture (PF) is a common complication of metastatic bone disease but is, rarely, the presentation of a primary bone tumor. We report a case of an 83-year-old man with a history of untreated prostate cancer, presenting with atraumatic fracture of his left femur after months of intermittent pains and weight loss. Radiographic workup revealed a lytic lesion suspicious for PF secondary to metastatic prostate cancer; however, initial core biopsy results were inconclusive for malignancy. A complete blood count with differential and complete metabolic panel was within normal limits. During surgical fixation and nailing of the femur, a reaming biopsy was performed as a repeat measure and revealed diffuse large B-cell lymphoma. Staging with positron emission tomography and computed tomography found no evidence of lymphatic or visceral involvement and chemotherapy was promptly initiated. This case highlights the diagnostic workup challenges for PF secondary to PBL, especially in the setting of concurrent malignancy. Because of the non-specific presentation of a lytic lesion on imaging associated with atraumatic fracture, we highlight PBL as an important diagnostic consideration.

## Introduction

The 2020 World Health Organization classification of tumors of bone classifies primary non-Hodgkin lymphoma of the bone as a neoplasm of malignant lymphoid cells in the bone with no lymph node involvement or other extranodal involvement (1). Originally termed “reticulum sarcoma of the bone” nearly a century ago, primary bone lymphoma (PBL) began to officially be histologically and cytologically recognized in the 1970s (2). However, PBL accounts for less than 1% of all lymphomas and less than 5% of all primary bone tumors (3, 4). Given its rarity, it has been difficult to employ a consistent definition and to fully understand the clinical and pathologic characteristics of this unique entity (5). On the basis of the available data, the average age of diagnosis is 45–60 years old with a slight predominance toward male patients (4).

Approximately 95% of diagnosed PBL is pathologically characterized as diffuse large B-cell lymphoma (PB-DLBCL) (4, 6, 7). Most cases present with localized pain, and nearly half of all cases are associated with localized swelling (8). Unlike other symptoms, pathological fracture (PF) is an unusual PBL complication (8). Commonly involved sites include the spine, lower extremity bones, and pelvis, with a predisposition toward the long bones at the site of the metaphysis and/or diaphysis (8, 9). Because of the non-specific features of this entity, the diagnostic process utilized in PBL contains many challenges. However, prompt treatment with rituximab and cyclophosphamide, doxorubicin, vincristine, and prednisone (CHOP)–based therapy in cases of PB-DLBCL has been shown to demonstrate favorable 5-year overall survival (OS) and progression-free survival (PFS) rates (7, 8).

Herein, we present an unusual presentation of PB-DLBCL as a PF in a patient with concurrent untreated prostate cancer. This case highlights the importance of considering PBL in the differential diagnosis for patients with a PF.

## Case presentation

**Figure 1:**
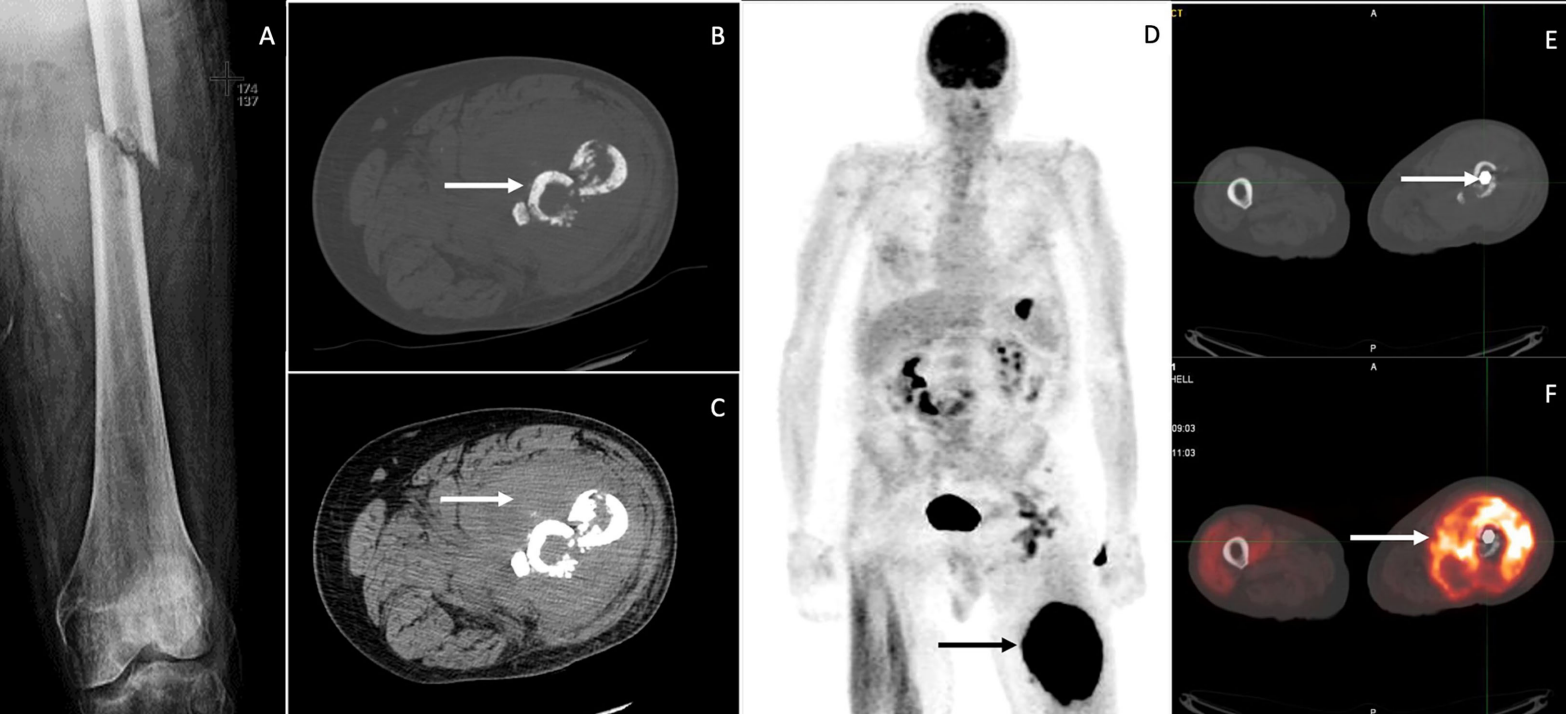
X-ray demonstrates displaced mid femoral pathologic fracture through a permeative lesion with improved alignment and diffuse osteopenia **(A)**. Axial images from CT of the left lower extremity showing a pathologic fracture involving the left proximal femur with sclerotic and fragmented bones (arrow in **(B)**, and a large heterogeneous soft tissue density mass surrounding the bone fragments (arrow in **(C)**. Whole-body image from the FDG PET/CT scan **(D)** shows intense hypermetabolism in the left thigh mass, consistent with neoplastic involvement. Other foci of increased uptake were thought to be non-neoplastic. The pathologic fracture has been internally fixed with an intramedullary nail (arrow in **(E)**, and the axial fused PET/CT image shows intense hypermetabolism in the mass surrounding the proximal femur (arrow in **(F)**.

An 83-year-old male Caucasian prison inmate with a past medical history of untreated prostate cancer presented to the prison hospital after spontaneous development of pain and weight-bearing limitation in the left leg. Over the previous 2 months, the patient reported intermittent sharp shooting pains in his left thigh and 10 pounds of unintentional weight loss. He denied experiencing any episodes of fever, chills, or night sweats, and the only other positive symptoms upon a review of systems included occasional constipation and diarrhea. Family and social histories were, otherwise, felt to be non-contributory. The physical exam was significant for tenderness to the left lower extremity at the superior femur with warmth but no bruising. Dorsalis pedis and posterior tibial pulses were palpable bilaterally. On musculoskeletal exam, the patient was able to move his toes and ankle without pain bilaterally but exhibited pain during left knee and hip range of motion tests. The range of motion at the left hip was completely inhibited by pain. He had a full painless range of motion in his right lower extremity. Complete blood count showed a decreased hemoglobin level of 10.5 g/dl with no other abnormalities; basic metabolic panel was notable for hypocalcemia to 7.9 mg/dl with all other electrolytes and creatinine within normal limits. Prostate specific antigen was 5.08 ng/ml. Left lower extremity x-ray showed an irregular 2.5-mm displaced left femur shaft fracture with a “moth-eaten” appearance consistent with PF (**Figure 1A**).

Computed tomography (CT) scan of the left lower extremity showed a comminuted fracture of the mid-femoral diaphysis with permeative appearance of the cortex at the level of the fracture. A large heterogeneous surrounding soft tissue enhancement was noted, representing a possible soft tissue lesion *vs*. hematoma (**Figures 1B–D**). Ultrasound-guided core needle biopsy did not reveal evidence of malignancy. Additional CT imaging of the chest/abdomen/pelvis was inconclusive.

**Figure 2:**
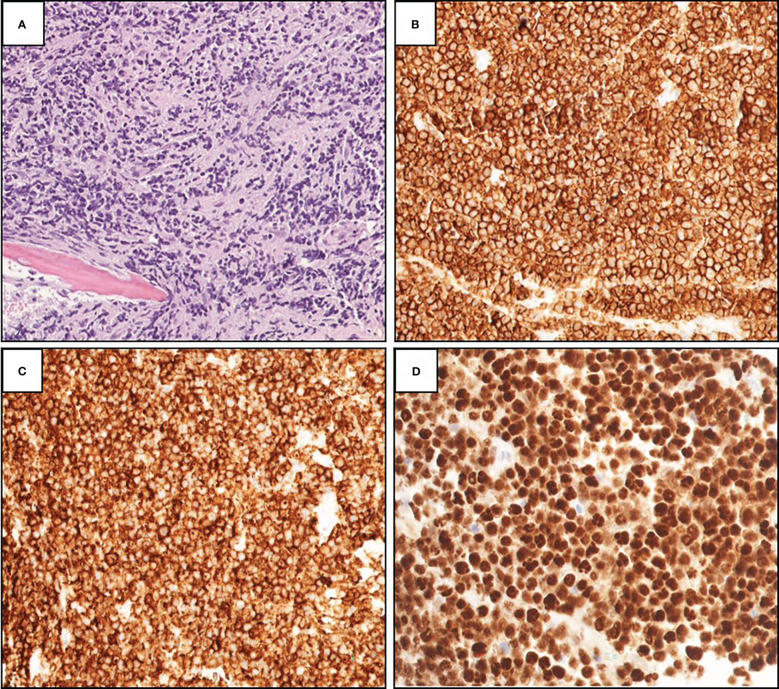
**(A)** Histopathological examination of showed focally necrotic and fibrotic diffuse lymphoid infiltrate surrounding bone trabecular (H&E, 200×). Immunohistochemistry showing strongly positive staining for CD20 **(B)**, 200×), CD10 **(C)**, 200×), and PAX-5 **(D)**, 200×).

Magnetic resonance imaging (MRI) was planned for the left leg, but the patient first underwent intramedullary nail fixation and reduction, during which a repeat and conclusive intraoperative left femur reaming biopsy was taken (**Figures 1E, F**). Histological and immunohistochemical (IHC) evaluation identified a focally necrotic, atypical lymphoid population positive for CD10 (>30%), CD20, CD45, PAX-5, Bcl-2 (>50%), and Bcl-6 (>30%) (**Figure 2**). Assessment *via* Ki-67 indicated a high proliferation rate at approximately 80%–90% in the viable areas. *In situ* hybridization was negative for Epstein–Barr virus. This established a diagnosis of DLBCL. Because of the presence of a solitary osseous lesion without extraosseous evidence of disease, the tumor was further classified as PB-DLBCL. The diagnostic assistance of MRI was no longer needed, so attention was turned toward treatment and staging.

Initial treatment was delayed because of the necessary post-operative recovery. A positron emission tomography (PET) and CT scan was performed, which revealed hypermetabolic activity of the mid-femoral bone at the site of the bone lesion (**Figures 1D–F**). Fluorodeoxyglucose (FDG) uptake of gastrointestinal sites prompted evaluation by esophagogastroduodenoscopy and colonoscopy, which showed no evidence of malignancy. The patient was consequently classified as having stage - 1E PB-DLBCL (10).

The patient completed five of six planned cycles of R-miniCHOP chemotherapy, consisting of 3-week intervals of rituximab (375 mg/m^2^), cyclophosphamide (400 mg/m^2^), doxorubicin (25 mg/m^2^), and vincristine (1 mg) all given intravenously on day 1 of each cycle (C) and oral prednisolone (40 mg/m^2^) on days 1–5. During this time, he noted a steady progression in his ambulatory status ultimately using a walker in his prison unit. Of note, vincristine was dropped after C2 from the regimen due to development of constipation, neuropathy, and palpitations. Pancytopenia was noted after C5 but resolved. Radiation therapy was planned with pending CT simulation, set to begin after completing chemotherapy. However, prior to his appointment for C6, the patient had a fall in his unit resulting in fracture of his right femur. He underwent subsequent right hip hemiarthroplasty which delayed C6. CT Simulation scan of the left hip was still done, which found incomplete response to chemotherapy. Repeat PET-CT was planned to re-stage, but the patient unfortunately developed septic shock secondary to right lower lobe pneumonia and died before any further diagnostic or treatment methods were employed.

## Discussion

PBL presents similarly compared to other bone malignancies. More than 90% of primary bone tumors are sarcomas including osteosarcoma, chondrosarcoma, and Ewing sarcomas (11). Unfortunately, both PBL and the sarcomas, as well as bone metastases, all typically feature pain and less frequently swelling, neurologic symptoms, and pathologic fracture (PF) (11–13). Thus, diagnosis requires radiographic characterization and pathology results. However, a primary bone tumor with no other concurrent tumors may entail a different diagnostic management plan than a new lesion in the setting of known metastatic disease. In our case, we were caught between these in the uncommon scenario where one solitary bone lesion of unknown etiology is found in the setting of a known primary, particularly one with a strong predisposition toward bony metastasis.

This case highlights the diagnostic challenge in delineating between primary and secondary bone malignancy in a patient with pre-existing prostate cancer and a newfound bone lesion, in this case presenting with PF. Pathological fractures are often indicative of advanced malignancy that has metastasized to the bone—namely, breast, prostate, lung, thyroid, and kidney cancers (14). Metastatic cancer spread involving bone results in PF in 20% of all cases of skeletal-related events (15). Metastatic prostate cancer was initially suspected in our patient, as most patients develop bone metastasis over the disease course (16), and most PFs are due to metastatic rather than primary bone cancer (17). Even within the scope of primary bone malignancy, PBL makes up less than 5% of cases (18). Hence, suspicion for PBL was exceedingly low at the onset of this case, but maintaining the adage that “a bone lesion with unknown etiology is a primary bone tumor until proven otherwise” provides an appropriate framework for approaching such a presentation (19). The diagnostic workup for PFs with unknown etiology outlined by Willeumier et al. suggests that, in the setting of a known pre-existing malignancy with no prior metastases, biopsy of the new unknown solitary lesion should be considered (19).

The most crucial diagnostic component in the evaluation of PF is the histologic assessment from biopsy (20). For initial biopsy of bone and soft tissue tumors, core needle biopsy is the standard alongside the incisional biopsy as compared to fine needle aspiration (FNA) (21). Percutaneous imaging–guided biopsy for pathologic bone lesions is especially difficult in the setting of PF (21). The well-known risks of an open procedure biopsy include infection, hematoma, and seeding of tumor cells among others (21). Moreover, a study by Afinowi et al. showed that 51% of reaming biopsies provided a positive diagnosis, whereas another 45% of cases of reportedly adequate samples resulted in false-negative results most commonly from samples associated with crushed artifacts or non-viable bone (P = 0.015) (22). The presence of crushed artifact and necrosis on biopsy samples from PBL lesions is well described in the literature and poses a serious challenge in obtaining diagnostic biopsy tissue samples (23). The usage of FNA to then perform flow cytometry has been discussed as an important diagnostic tool that may alleviate the issue of crushed artifact and necrosis associated with PBL (24, 25). In our case, without conclusive results from the initial biopsy, we decided to perform reaming biopsy during the intramedullary nailing (IMN) fixation and reduction procedure. Although Gusho et al. pushed against a role for reaming biopsies during IMN fixation and reduction in known metastatic bone disease patients due to frequent inadequacy of samples and little impact on clinical management, this dual diagnostic/therapeutic management step is poorly described in patients with a known primary and solitary new lesion but no prior metastases (26). In such patients, this case demonstrates that performing a reaming biopsy during IMN fixation may help get additional information regarding new mutations.

This case also illustrates multiple similarities between prostatic metastasis and PBL, particularly on imaging. Metastatic prostate cancer and PBL both frequently present in the vertebral column, most commonly in the lumbar spine (6, 27). The rates of long bone involvement in PBL and metastatic prostate cancer are also significant, at 23% and 15%, respectively (6, 28). Up to 22% of metastatic prostate cancer in bone results with PF (29). However, PF in PBL is less common and has accounted for approximately 10%–15% of presentations in previous studies (30–32).

The typical radiographic and CT characteristics that indicate pathologic rather than stress fractures include aggressive (rather than benign) periosteal reaction, a lytic or permeative marrow pattern, and the presence of a soft-tissue mass (33). Although we were unable to use MRI, it is a more suitable imaging modality for this purpose, with characteristic findings for PF including homogenous T1-hypointense signal abnormality at the site of fracture due, in part, to the tumor itself and potentially, in part, to edema and bleeding at the site (33). Whereas prostatic metastases are typically osteosclerotic or osteoblastic in appearance on plain film radiography, PBL and the sarcomas predominantly exhibit an osteolytic pattern with soft tissue extension (34–36). Moreover, PBL and Ewing Sarcoma are known to cause periosteal reactions (35, 36). However, prostatic bony metastases were found in one study to present as osteolytic themselves 16.4% of the time (37). Likewise, mixed blastic-lytic lesions are a possible radiological presentation of both prostatic metastases and PBL (34, 35). Last, one significant finding on CT was the surrounding soft tissue enhancement. With a hallmark of PF appearance on CT, soft tissue enhancement can represent extraosseous or soft tissue extension of a bony tumor. However, although this phenomenon is noteworthy for PBL, it has also been found in up to a third of prostatic metastases (35, 38). Thus, osteolytic appearance on plain film radiography may be of more clinical utility than soft tissue extension in distinguishing between these two entities.

Although MRI is an excellent diagnostic tool in the workup of unknown bone malignancy, it was ultimately not needed upon diagnosis with histopathology and staging *via* PET. PET-CT scanning is quickly gaining interest as a tool for initial characterization, staging, biopsy selection, evaluation of treatment response, and surveillance of tumor recurrence (39, 40). This is largely due to the high sensitivity of PET-CT imaging in the detection of PBL lesions, with a sensitivity of 90%–100% (4, 39, 40). PET-CT imaging can be particularly valuable in the staging of PBL due to its ability to evaluate for the presence of whole-body disease involvement, as opposed to nuclear MRI or CT imaging (41). Furthermore, repeat post-treatment imaging with a PET-CT scan can evaluate the response to treatment through the measurement of metabolic activity using standard uptake value quantification (39, 40).

IHC evaluation of diagnostic material was positive for DLBCL Germinal Center B-cell (GCB), which has a better prognosis and is commonly found in PB-DLBCL (7, 8). A study by de Groen et al. evaluated different OS/PFS rates in patients with PB-DLBCL, NO-DLBCL, polyostotic-DLBCL, and disseminated-DLBCL using histologic, IHC, FISH, gene expression profiling, and targeted next-generation sequencing (5). Their study revealed that 68% of stage I/II PB-DLBCL tumors had at least one mutation in B2M, EZH2, IRF8, and TNFRSF14 compared with fewer mutations in stage I/II NO-DLBCL tumors (5). Furthermore, PB-DLBCL had significant differences in immune surveillance gene expression compared with NO-DLBCL (5). Their study also revealed that stage I/II PB-DLBCL had a statistically significant greater difference in OS/PFS versus stage I/II NO-DLBCL (5). These distinct differences in gene expression and outcomes between these two classifications of DLBCL provide additional evidence that PB-DLBCL should be treated as a unique entity and may be useful in the differentiation of PB-DLBCL and NO-DLBCL with osseous involvement.

Although surgery plays an important role in bone stabilization and resolution of fracture, PBL treatment typically consists of anti-CD20 (rituximab) and CHOP based therapies, with the possible addition of radiotherapy (RT) if necessary (8). Compared with NO-DLBCL, patients with PB-DLBCL have favorable 5-year OS rates, ranging from 60% to 95% (mean OS, 82%) (7, 8). A study by Li et al. demonstrated that 90% of PB-DLBCL has a GCB-like immunophenotype that is associated with better prognosis compared with non-GCB–like immunophenotype (7). However, despite better OS and PFS in PB-DLBCL, pre-treatment characteristics and events such as PF are associated with worse prognostic outcomes (8, 9). A multicenter study by Govi et al. demonstrated that initial treatment of PF with RT in patients with DLBCL experienced worse OS compared with patients treated initially with chemotherapy (5-year OS: 22 ± 14% versus 64 ± 9%, P = 0.007) (9). Nevertheless, studies evaluating the role of RT in PB-DLBCL have produced mixed results.

One severe limitation in our approach to this case was poor access to MRI, normally an essential aid to diagnosis of any bone lesion suspicious for cancer. Likewise, our initial ultrasound-guided core needle biopsy was unhelpful, the inconclusive results of which may be insufficient amount of diagnostic material. However, we were able to obtain an adequate sample to establish our diagnosis from the reaming biopsies. Furthermore, lack of interim or routine surveill

63ance PET-CT (i.e., every two cycles as recommended by Zhang et al.) hindered our treatment plan, as the patient was ultimately not showing much response to the r-mini CHOP regimen (42). However, this result may have been further complicated by having to stop vincristine due to adverse effects.

## Conclusion

Here, we summarize our experience with managing PB-DLBCL presenting with PF in a patient with a synchronous untreated prostate cancer and provide a comprehensive review of literature on this topic, with emphasis on the diagnostic challenges and use of reaming biopsy during fracture stabilization to make the diagnosis. Although PBL rarely presents with PF and even more rarely is synchronous with another primary tumor, it should be considered as a possible primary bone tumor in the differential diagnosis for PF.

## Data availability statement

The original contributions presented in the study are included in the article/supplementary material. Further inquiries can be directed to the corresponding author.

## Ethics statement

Written informed consent was obtained from the individual(s) for the publication of any potentially identifiable images or data included in this article.

## Author contributions

Study conception and design: PoB, AY, and KL. Manuscript preparation/drafting: PoB, ZH, AY, BG, RA, DW, PeB, AR, TM, PM, JM, and KL. Expert review: KL. All authors contributed to the article and approved the submitted version.
